# Agent Inaccessibility as a Fundamental Principle in Quantum Mechanics: Objective Unpredictability and Formal Uncomputability

**DOI:** 10.3390/e21010004

**Published:** 2018-12-21

**Authors:** Jan Walleczek

**Affiliations:** Phenoscience Laboratories, Novalisstrasse 11, Aufgang F, 10115 Berlin, Germany; walleczek@phenoscience.com

**Keywords:** ontological quantum mechanics, objective non-signaling constraint, quantum inaccessibility, epistemic agent, emergent quantum state, self-referential dynamics, dynamical chaos, computational irreducibility, undecidable dynamics, Turing incomputability

## Abstract

The inaccessibility to the experimenter agent of the complete quantum state is well-known. However, decisive answers are still missing for the following question: What underpins and governs the physics of agent inaccessibility? Specifically, how does nature prevent the agent from accessing, predicting, and controlling, individual quantum measurement outcomes? The orthodox interpretation of quantum mechanics employs the metaphysical assumption of indeterminism—‘intrinsic randomness’—as an axiomatic, in-principle limit on agent–quantum access. By contrast, ontological and deterministic interpretations of quantum mechanics typically adopt an operational, in-practice limit on agent access and knowledge—‘effective ignorance’. The present work considers a third option—‘objective ignorance’: an in-principle limit for ontological quantum mechanics based upon self-referential dynamics, including undecidable dynamics and dynamical chaos, employing uncomputability as a formal limit. Given a typical quantum random sequence, no formal proof is available for the truth of quantum indeterminism, whereas a formal proof for the uncomputability of the quantum random sequence—as a fundamental limit on agent access ensuring objective unpredictability—is a plausible option. This forms the basis of the present proposal for an agent-inaccessibility principle in quantum mechanics.

## 1. Introduction

The fast rising interest in ontological quantum mechanics has brought to the fore again the problem of the fundamental limits of experimenter agency in quantum mechanics. For example, the physical consistency of de Broglie-Bohm (dBB) theory [1,2,3,4] and Bohmian mechanics [5,6,7,8], as well as recent quantum models within the ontological model framework [9,10,11,12], depends strictly on the imposition of a limit on agent access to nature. However, what governs the physics of ‘agent inaccessibility’? How and why does nature prohibit the experimenter agent from having unlimited access to reality at the level of the quantum? Is the universe “fine-tuned” against agent access to the quantum state? What is the difference between ‘agents’ and observers’ in relation to quantum inaccessibility? Finally, if agent inaccessibility is fundamental, then what is the ontological status of inaccessible quantum states?

The specific choice of an answer to these foundational questions strongly constrains the plausibility of any type of quantum-ontological formalism, whether for ψ-ontic or ψ-epistemic interpretations [9,11], including for quantum models that involve globally deterministic constraints [13,14,15,16,17], such as those exploring the possibility of an emergent quantum mechanics (e.g., see the Special Issue on Emergent Quantum Mechanics in the *Entropy* journal). Critically, this suggests that an *informal* principle like ‘agent inaccessibility’ can decide whether—or not—a *formal* quantum model, or related mathematical theorem, might be physically realistic in view of the known record of quantum observations in the laboratory. In terms of advancing a physical account of EPR-type quantum correlations, for example, how to assess whether a proposed quantum formalism is prone to causal-paradox formation? The ineliminable dependence—apparently—of the respective answers upon an informal agent-centric notion should cause concern and motivate the development of a model or theory of the physics of agent inaccessibility.

The present work considers an agent-inaccessibility principle (AIP) as a fundamental principle in quantum mechanics. This analysis adopts the standard assumption that individual quantum detection events are *objectively* unpredictable, i.e., unpredictable by *any* experimenter agents. In search of an explanation for quantum unpredictability, three distinct physical scenarios will be compared, as captured by the concepts of (i) intrinsic randomness, (ii) effective ignorance, and finally (iii) absolute or objective ignorance (see Section 5). The latter concept introduces the possibility of an in-principle limit for agent inaccessibility based upon formal uncomputability and objective unpredictability. As a definition of objective unpredictability, and of objective non-signaling, in quantum mechanics, three types of uncomputability will be considered, all of which are based upon self-referential relations: (i) uncomputability due to the impossibility to know initial conditions with infinite precision, as in dynamical chaos, (ii) uncomputability due to ‘computational irreducibility’ [18,19], and (iii) uncomputability due to the halting problem as specified in the Church-Turing thesis [20,21]. Regarding the latter concept, the term ‘Turing incomputability’ will also be employed in this article. Next, without adopting an AIP, how could an ontological quantum theory be physically realistic?

## 2. Many-World and Single-World Quantum Interpretations

A well-known instance of an ontological quantum interpretation that might—possibly—do without an AIP is Everett’s many-worlds (MW) interpretation [22,23]. The problem of (non-signaling) agent access is circumvented in the MW interpretation by branching—upon the agent’s measurement of the quantum state—into parallel world ontologies. However, in the MW interpretation, the agent is prohibited from accessing any world ontology but the agent’s own, which is, again, a notion of agent inaccessibility, and one that lacks a *physical* explanation in the MW proposal. For many-interacting-worlds interpretations, see References [24,25,26]. For any single-world (SW) quantum ontology, in particular, such as dBB-theory and Bohmian mechanics, but also for theories involving time-symmetric ontologies, the adoption of an AIP appears to be strictly required in view of possible violations of the non-signaling theorem of quantum mechanics (for an overview see, e.g., Reference [27]). Consequently, the question of whether an experimenter agent can access, predict, compute, and control, quantum information, e.g., as involved in EPR-type quantum correlations during tests of Bell’s inequality [28], is crucial for assessing the plausibility of any proposed quantum formalism, whether the formalism posits (local) retrocausality [12,13,14,15,16,29,30,31,32], or nonlocality [1,2,3,4,5,6,7,8], including in the development of an emergent quantum mechanics (e.g., Reference [17]).

The target of the present analysis will be SW quantum interpretations in relation to agent inaccessibility. A defining feature of any SW interpretation, whether it is an operational or an ontological one, is that “…from the viewpoint of an agent who carries out a measurement, this measurement has one single outcome”, as was explained by Frauchiger and Renner [33] in the context of their recent argument against the self-consistency of quantum theory due to self-referential relations—in Wigner’s friend paradox—between multiple experimenter agents. The significance of the phrase “from the viewpoint of an agent” concerns the additional question—in relation to the single outcome in a SW interpretation—of whether a quantum detection event, e.g., a ‘spin-up’ observation by an agent in the laboratory, does—or does not—constitute an “objective fact” of nature. For different criticisms of the argument by Frauchiger and Renner [33], see References [34,35,36].

## 3. Restricting Agent Access to Ontological Quantum States and Quantum Information

The physical plausibility of SW realist quantum theories, including those based upon nonlocal or retrocausal quantum ontologies, has long been recognized to depend strictly on the assumption that an ontic state (λ) exists whose exact properties are inaccessible to, and hence unobservable by, an experimenter agent. For example, in reference to a time-symmetric quantum ontology, Leifer and Pusey [12] have found that regarding the “…exact ontic state… we cannot actually construct an experiment that would reveal it”. An example from a wholly different context is decoherence theory, where “…definite, classical pointer states are selected in the interaction between environment and system” as Zwolak and Zurek [37] explained. There, constraints on agent access are also adopted, of course, and it was noted by these authors [37] that “…a world where objective information is present is also a world with quantum information inaccessible to all but the most encompassing observer”.

The above examples serve as reminders that agent inaccessibility is a central and unavoidable concept in quantum mechanics. That is, the existence of inaccessible quantum information is assumed in diverse quantum-theoretical contexts, and ontological quantum mechanics must typically posit the existence of an ontic state λ whose exact properties are experimentally unobservable. Is it physically feasible, however, that strictly inaccessible, i.e., unobservable, ontic states may—in fact—exist? What is the ontological status of a property or information that does *exist* but that could not be accessed and predicted either *in-practice* or *in-principle*?

### 3.1. On the Reality of an Indefinite Quantum Ontology: Contextuality and Relationality

An ontological regime whose exact properties are unobservable because they cannot—actually—be revealed experimentally, will be called an indefinite ontology. The term ‘indefinite’ was chosen as a neutral term in reference to an ontological state prior to its measurement, whether or not that state might possess relational or contextual properties. For clarification, regarding an indefinite (possibly relational or contextual) ontology, the question is not whether a property exists “when no one is looking”, but whether some property, or value, exists that cannot be predicted by any amount of “looking”, i.e., by any local or nonlocal tests, including computer simulations, prior to performing the actual measurement. That question is closely tied, of course, to the well-known fact that the predictions of orthodox quantum mechanics are wholly incompatible with the (naïve realist) notion of pre-existing quantum properties, i.e., with the false notion that quantum states may possess (non-contextual) definite properties or values prior to, and independent of, their measurement. Put generally, a non-contextual (non-relational) property of an ontological system is one whose outcome state or value is entirely independent of whether, or how, the property is measured by the agent. It is well-known that Einstein, Podolsky, and Rosen, first introduced a definition of definite, non-contextual ontic states in relation to the problem of “action-at-a-distance” in quantum mechanics—the concept of “the elements of reality” [38]. To be sure, quantum ontologies that could be consistent with orthodox quantum predictions must—by contrast—possess value-indefinite properties, thereby allowing consistency with the physical demands of the theorem by Kochen and Specker [39].

Again, the term ‘indefinite ontology’ is employed because, prior to any measuring interaction, ontic state λ exists in an indefinite state, i.e., a state whose exact value is not accessible, computable, or predictable; by contrast, again, a definite, i.e., measurement-independent, state is one whose value could—in principle—be accessed in nature by the experimenter agent. Importantly, using the present terminology, a quantum-measurement process entails the transformation of an indefinite ontic state (IOS) into a definite ontic state (DOS). Consequently, the standard measurement problem of quantum mechanics is recast as the problem of how to explain, and how to conceptualize, an IOS–DOS transition event. By that definition, a contextual, or relational, ontology is simply one that is governed by IOS-DOS transitioning during the (dynamical) process when the agent performs a measurement upon the quantum state as defined by a particular ontological quantum model.

For explanation, take a typical, experimentally generated quantum random sequence. In the orthodox interpretation of quantum mechanics, each individual random event is presumed to be objectively unpredictable as a function of quantum indeterminacy (see also Section 5.1.1). However, and this is the main proposal of the present analysis, there may be another option for explaining quantum unpredictability—an explanation that is compatible with the presence of an underlying ontology. In the ontological option, each one of the individual quantum detection events that together constitute a quantum random sequence, existed—prior to the measurement-dependent DOS transformation—in the form of an IOS, which is a state possessing value-indefinite properties (e.g., see References [40,41,42]); only the actual measurement interaction induces the IOS-DOS transition which results in the definite value of the measured ontic state. The present work considers the proposal that an indefinite, likely contextual, ontic state λ represents either (i) an effectively uncomputable element in the weak option of ‘effective ignorance’ (Section 5.2), or (ii) an objectively uncomputable element in the strong option of ‘objective ignorance’ (Section 5.3).

Notably, contextuality might represent the “non-information-theoretic kernel” of quantum theory, Koberinski and Müller [43] have suggested recently, and that therefore contextuality could be a genuine physical (ontological) feature of a possible quantum reality. With respect to contextuality as a “genuine physical feature”, the investigators cited Fuchs [44] who had also expressed the similar hope earlier that the “…distillate that remains—a piece of quantum theory with no information-theoretic significance—will be our first unadorned glimpse of ‘quantum reality’.” Related to that suggestion, the following idea is here pursued also: what Koberinski and Müller [43] have referred to as the “non-information-theoretic kernel” of quantum theory may refer directly to the uncomputable ontological features that are the topic of the present article (compare Section 6).

### 3.2. The Inaccessible Universe and the Limits of Science

In addition to an ontology being contextual or relational, further important questions are (i) whether the ontology is nonlocal [1,2,3,4,5,6,7,8], locally time-symmetric [12,13,14,15,16,29,30,31,32], or locally time-asymmetric [45,46,47], and (ii) what an experimenter agent can know exactly about a given quantum ontology. For example, in the case of the above-mentioned, time-symmetric ontological model, Leifer and Pusey [12] have noted that the exact ontic state λ—although it “…may be unknown to the experimenter”—“…is in principle knowable”. If so, then in what specific manner? To address questions such as these, the present work proceeds by investigating this general question: What is the ontological status of an empirically inaccessible regime of physical reality? 

An objective limit on access to the nature of reality is, of course, anathema to the goals of the project of modern science. Science is thought to be about an understanding of reality based upon the capacity to measure, predict, compute, and control. By contrast, the revolutionary discovery of the fundamental quantization of matter and energy has long been held to imply that—at its smallest dimensions—the universe is intrinsically random, which—from the start—prevents an agent from accessing, predicting, and controlling, individual measurement outcomes. This is, of course, the standard position known as the orthodox interpretation of quantum mechanics. With the advent of ontological quantum mechanics, however, science started to consider the possibility that (ontic) “elements of reality” might exist—at the quantum level—in a form that is both compatible with (i) determinism as well as with (ii) contextuality and single-event unpredictability. However, prior to answering the question of how a fully deterministic system may produce outcome states that are unpredictable and uncomputable as a matter of principle, three related issues will be considered first: (i) the no-hidden-variables theorems in quantum mechanics (Section 3.2.1), (ii) the concept of agent-inaccessible variables (Section 3.3), and (iii) the definition of the experimenter agent (Section 4).

#### 3.2.1. On No-Hidden-Variables Theorems in Ontological Quantum Mechanics

As a way to begin to frame the above question of unpredictability in deterministic systems, the ontological status will be reviewed briefly of the variables called ‘hidden’ in the original formulation of an ontological quantum theory, namely in dBB-theory [1,2,3]. The present analysis argues that the introduction of the ‘hidden variable’ (HV) marked a turning point, not only for quantum physics, but for modern science in general. That is, if proven valid, the HV-concept necessitates the introduction of a radical limit for science: the idea that an inaccessible, or hidden, ontology of nature exists, which is beyond the scientific method to measure, predict, compute, and control (compare Section 6). Importantly, it is the very HV-concept which may ensure that a model of quantum reality could be free from causal-paradox formation, by prohibiting, for example, superluminal signaling and communication, in the typical thought experiments that envision physical inconsistencies due to unorthodox ontological propositions such as nonlocality (e.g., [27]). The opposite and orthodox view has long been defended by those who have employed the traditional “no-hidden-variables” theorems, i.e., the no-go theorems against the physical plausibility of, for example, dBB-theory and Bohmian mechanics (see also Section 5.1).

For critical views arguing against standard interpretations of no-hidden-variables theorems see, for example, Mermin [48], Maudlin [49,50], Lazarovici et al. [51], Passon [52], Tumulka [8,53], Norsen [6,54], Palmer [47], De Gosson [55], Wharton [13,32], Adlam [14,15], Ghadimi et al. [26], Khrennikov [56,57], Hiley and Van Reeth [58], Flack and Hiley [59], and Walleczek [60]. After performing a careful analysis, Gisin [61] noted recently that “…Bohmian mechanics is deeply consistent”, and he remarked that “Bohmian mechanics… could inspire brave new ideas that challenge quantum physics.”

### 3.3. Hidden-Variables in Quantum Mechanics are Agent-Inaccessible Variables

The concept of the HV in quantum mechanics was introduced by David Bohm [1,2]. In original dBB-theory, the mathematical formalism refers to hypothetical ontic elements such as the quantum potential [1,2,3,4]. Crucially, to avoid any misunderstanding, it should be mentioned that dBB-theory, which has also been developed in another context as Bohmian mechanics [5,6,7,8], is not a classical, ontological theory, but an ontological theory manifesting entirely non-classical properties, including nonlocality. The term ‘hidden’ usually explains this in Bohm’s theory: no measurement can be performed that might reveal exact information about the ontic state in a way that allows an experimenter agent to controllably direct nonlocal information transfers. For example, Holland [4] commented that “…the quantum potential implies that a certain kind of ‘signaling’ does, in fact, take place between the sites of distantly separated… particles in an entangled state”, but that this “…transfer of information cannot, however, be extracted by any experiment which obeys the laws of quantum mechanics”. More recently, Valentini [62] had also remarked that this “…information flow is not visible at the statistical level”. Walleczek and Grössing [27] have clarified the point that this nonlocal quantum information transfer must not be understood as information transfer in any communication-theoretic sense. That is, for an ontological quantum theory, such as dBB-theory, which is both contextual and nonlocal (e.g., [48]), the adoption of an AIP—as an informal non-transfer-control theorem in Reference [27]—prohibits access to, and the instrumental control of, nonlocal information transfers for the purpose of sending superluminal (Shannon-type) signals, or messages, between sender and receiver, while—at the same time—allowing the presence of non-Shannon signals [27]. Please note that the term ‘hidden signaling’ has also been used recently, for example by Bendersky et al. [63], in reference to the concept of non-Shannon signaling [27].

In summary, in a quantum theory such as dBB-theory, the HV indicates the presence of an indefinite ontological element in the theory (i.e., ontic state λ) whose exact value cannot be accessed, predicted, or controlled (e.g., a spin property). That is, again, the HV-concept refers to an unobservable property, not merely to one that is unobserved, and—as a consequence—it cannot be controlled by an observing agent (see Section 3.1). Therefore, John Bell [64], for example, noted that “The usual nomenclature, hidden variables, is most unfortunate”, and he proposed that “Perhaps uncontrolled variable would have been better, for these variables, by hypothesis, for the time being, cannot be manipulated at will by us.” The present work continues in the spirit Bell’s understanding that a variable called ‘hidden’ represents an uncontrollable variable, i.e., a variable that “cannot be manipulated at will by us” [64]—an *agent*-inaccessible variable using the present terminology. Therefore, before proceeding any further, a definition should be given for what constitutes an ‘agent’—as opposed to an ‘observer’—in quantum physics and for science in general. How to define the experimenter agent to begin with?

## 4. Defining the Experimenter Agent

In the particular context of assessing the role of the agent in relation to the non-signaling theorem, John Bell [65] insisted that needed is at least “…a fragment of a theory of the human being” to be able to address the question of whether or not “we can signal faster than light?”. Put differently, Bell requested having a partial theory, at least, of what defines agency in the context of quantum physics. Specifically, the definition should be relevant, as Bell [65] requested, to the question of who “we think we are, we who can make measurements, we who can manipulate external fields, we who can signal at all, even if not faster than light?”. In the context of Bell’s theorem, a consistent understanding of the notions of agent-dependent versus agent-independent signaling—in terms of Shannon versus non-Shannon signaling—is available from the above-mentioned analysis that applied the operational framework of Shannon’s mathematical theory of communication to answer Bell’s questions regarding the valid interpretation of the non-signaling theorem [27]. 

As was described by Walleczek and Grössing [27], an experimenter agent is not merely an observer in the world but is an entity capable of acting in the world in the pursuit of goals, such as (i) in setting-up an experiment for the purpose of asking questions of nature, or (ii) in selecting specific measurement settings (for details see Section 4.3). However, the continuing lack of a model of, or of a theory for, the experimenter agent in quantum physics, and in science in general, impedes making progress towards understanding the foundations of quantum mechanics. The present work suggests that the success to counter the no-go theorems against the possibility of an ontological quantum mechanics also depends (i) on the particular model of the experimenter agent, and (ii) on an understanding of the distinctive role of an AIP in ontological and deterministic interpretations of quantum mechanics (see Section 3).

### 4.1. The Quantum Measurement Problem

For a long time, the observing agent was considered in the context only of the familiar quantum-measurement problem, especially vis-à-vis collapse-type interpretations such as the Copenhagen interpretation (for an introduction see, e.g., Reference [66]). In recent years, however, the distinct significance of the notions of observation *versus* agency has been recognized well beyond the issue of collapsing the wave function. It is increasingly understood that the concept of the experimenter agent is central to any plausible SW interpretation of ontological quantum theories, not only for ψ-epistemic or purely operational interpretations, such as for quantum Bayesianism [67]. The present work, therefore, seeks to establish a minimum framework, one that is capable of addressing the question of the limits of ‘observer agency’ in the context of new ontological perspectives for quantum physics. For example, as was described above, traditional assumptions and theorems such as nonlocality, contextuality, free choice, and non-signaling, need not necessarily contradict the existence of certain quantum ontologies. Importantly, the non-contradiction, i.e., the theoretical consistency, of permissible ontologies, such as in the measurement problem as captured by the concept of IOS-DOS transitioning described in Section 3.1, depends on the validity of an AIP in relation to a given quantum formalism. In light of an AIP, who or what is the experimenter agent?

### 4.2. An Early Definition of the Experimenter Agent: “Maxwell’s Demon”

An early notion of the experimenter agent was introduced into physics by James Clerk Maxwell [68]. To review briefly, in Maxwell’s thought experiment, an intelligent being or agent was proposed to be capable of lowering the entropy of a “closed” physical system. This being or agent became known of course as ‘Maxwell’s demon’—a ‘demon’ because of the apparent supernatural powers to observe, and act in, the world. The term ‘super-natural’ is used to characterize the kind of exceptional demon agency which Maxwell (falsely) presumed to be “free” from known natural constraints, such as from the Law of Energy Conservation. In short, Maxwell’s agent adopts therefore an isolated and quasi-transcendent position towards the rest of the physical universe (see also Section 5.2). In this pre-quantum thought experiment, the feat of entropy reduction is achieved by micro-causal interventions of the observing demon-agent who is granted unlimited access to, and predictive control over, the relevant microphysical processes of the targeted system: first, the agent observes microscopic events, and, second—based on observational knowledge—selectively acts upon the physical system so that the system becomes increasingly ordered. That is, in Maxwell’s thought experiment, knowledge-based agent interventions can predictably counter the intrinsic tendency of the closed system to spontaneously disorganize. The problem of the apparent violation of the Second Law of Thermodynamics by the “ordering agent influence” was, of course, first resolved formally by Szilard [69]. It is noteworthy in the present context that elements of Szilard’s proof assisted in the development of von Neumann’s mathematical foundations of quantum mechanics [70]. The point will be made next that, despite the known shortcomings, the concept of Maxwell’s demon captures key features that are still relevant to recent definitions of the experimenter agent (Section 4.3).

### 4.3. Recent Definition of the Experimenter Agent: “Epistemic Agency”

Already in the early concept of Maxwell’s demon were implicit two distinct capacities which continue to be employed in recent definitions of the experimenter agent: (i) the capacity of the agent to observe and to obtain knowledge (the epistemic dimension), and (ii) the capacity of the agent to act in the world in the pursuit of a goal (the agentic dimension). Hence, the term ‘epistemic agent’ can be used synonymously with the term ‘observing agent’. The following informal definition for epistemic agency was introduced previously [27]:

“Agency is generally defined as the capacity of humans or other entities to act in the world. Put differently, an agent is defined initially by possessing the capacity to influence causal flows in nature. By prefacing “agent” with the term “epistemic”, attention is drawn to the fact that a complete definition of agency represents more than the mere “capacity to influence causal flows”: an agent possesses knowledge-based, i.e., epistemic, capacity for predictably directing, and redirecting, causal flows, and thus for directing, and redirecting, information flows as well. That is, an epistemic agent holds the power to (statistically) control physical activity based upon an ability to predict the outcome of specific actions on targeted processes in reference to a known standard or goal. In short, an epistemic agent thus manifests in the world a genuine source of operational control”.

Importantly, the above definition of ‘operational control’—as a criterion for epistemic agency—ensures that entities other than human systems, such as artificial devices implementing goal-driven control systems, including devices and algorithms capable of computation and message communication, qualify as complete epistemic agents. Finally, in contrast to the pre-quantum conception of the agent in Maxwell’s thought experiment (Section 4.2), after the quantum revolution, from the perspective of the agent as an effective actor in the world, agent inaccessibility is now characterized by the denial of operational control in relation to an inaccessible quantum regime of nature. For example, ‘t Hooft [71] noted recently also that what “…distinguishes quantum systems from classical ones is our fundamental inability to control the microscopic details of the initial state…”. Critically, in the present proposal for an AIP, the measure of ‘operational control’ is the computational accessibility and predictability of physical processes by the agent. This raises the all-important question of exactly how nature—after the quantum revolution—prohibits (computational) access to the experimenter agent in a way that the purely classical world view—apparently—could not.

## 5. How does Nature Prohibit Access to the Experimenter Agent?

No scientific consensus exists concerning the question of how nature denies unlimited access to the experimenter agent of quantum states and quantum information. Entirely different physical explanations are on offer—as part of different quantum interpretations—regarding how nature limits agent access to quantum states or information and, therefore, how nature prohibits the prediction, and operational control by epistemic agents of individual quantum measurement outcomes. As was noted already, pre-quantum, classical, physics, by contrast, knows of no fundamental limits regarding agent access to nature (compare Section 4.2).

In the textbook, SW operational interpretation, which is orthodox quantum mechanics, it is the metaphysical assumption of ‘intrinsic randomness’, i.e., ‘quantum indeterminism’, which fundamentally limits the powers of the agent to predict the value of a single measurement outcome (see Section 5.1). By contrast, an ontological quantum theory, such as dBB-theory, typically derives its constraint on quantum predictability from the technological inability of the experimenter agent to collect complete information about initial conditions (see Section 5.2). These opposing explanations are frequently discussed in terms of in-principle versus in-practice limits of agent-access to quantum systems. It is often presumed that an in-principle limit to agent-quantum access can only be posited in the case of operational quantum approaches, whereas only an apparently weaker, in-practice limit is available for ontological quantum mechanics.

The present work introduces a third option: the possibility of an in-principle limit for ontological quantum mechanics based upon self-referential dynamics which may produce outcome states whose predictability would require either (i) access to infinitely precise knowledge about initial conditions and/or (ii) the availability of infinite computational resources (see Section 5.3). In the following, the three distinct options will be compared, whereby each one, albeit based on completely different physical assumptions, seeks to explain how nature prevents the agent from computing, predicting, and controlling, individual quantum events. First, the standard position of ‘universal indeterminism’ will be briefly discussed and criticized in Section 5.1.

### 5.1. Orthodox Quantum Mechanics: “Universal Indeterminism”

In orthodox quantum mechanics, the assumption of ‘intrinsic randomness’ serves as an absolute barrier to agent knowledge at the quantum level. Importantly, in the orthodox interpretation, the observed randomness is viewed as an *a priori* property of nature herself, e.g., prior to any additional physical constraints involving the agent. Remarkably, in universal indeterminism, a single random event can initiate an entirely new causal chain—apparently “out of nothing” (e.g., [72]). Nevertheless, and this—again—is the remarkable feature, the detection, for example, of a single ‘spin-up’ event by the measuring apparatus manifests a classical (pointer) state from which may propagate new causal flows, such as those triggering the formation of new biophysical events during sensory perception in the agent who observes the ‘spin-up’ measurement outcome. However, the question of what the exact nature might be of that initiating event, i.e., the question of ‘what is a quantum?’, is not addressed—famously—in the orthodox interpretation, and therefore, Plotnitsky [73], for example, has noted that “…quantum objects are seen as indescribable and possibly even as inconceivable”, in the indeterministic interpretation of textbook quantum physics.

**Figure 1:**
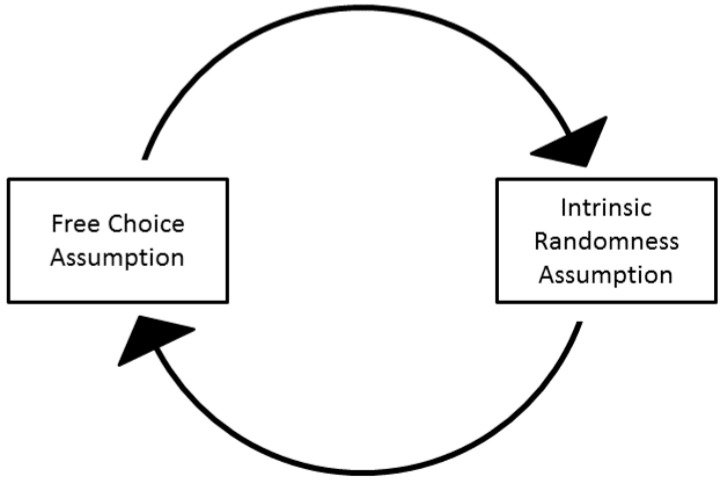
Quantum super-indeterminism [60]. The shortcomings of the orthodox view, which are revealed by the simple concept of super-indeterminism, in the attempt to prove, or justify, the metaphysics behind quantum indeterminacy, are recognized increasingly. The fallacy of circular reasoning is illustrated in Figure 1, which arises from the use of the intrinsic randomness assumption in support of the free choice assumption, which—in turn—rationalizes the presumably “free” selection of measurement settings. Bera et al. [76], for example, have confirmed the fact of ‘super-indeterminism’ by noting that there is indeed present “…an unavoidable *circulus vitiosus*” in any tests for true randomness, because any available tests for “…the indeterministic character of the physical reality” must presume that “…it is, in fact, indeterministic.” Similar arguments have been put forth by, and prior developments were summarized in, Landsman [77].

What is problematic, however, is that the very same indeterminism, or quantum randomness, which already serves as an absolute limit on agent knowledge, is often—at the same time—held to be the source also of the free-willed agency of the experimenter as in the free-will theorem by Conway and Kochen [74,75]. This is the exact opposite of being the source of a universal constraint. How could this be? How could one and the same (quantum) randomness be the source of both (i) objective chance and (ii) free-willed agent control of physical events in the world, such as freely selecting a measurement setting? This self-contradictory view, which has previously been captured in the concept of quantum super-indeterminism (see Figure 1), has long obscured insight into the plausibility of those no-go arguments against the possibility of ontological quantum mechanics which are based upon the freedom of choice of the experimenter agent (for an overview see Walleczek [60]).

**Figure 2:**
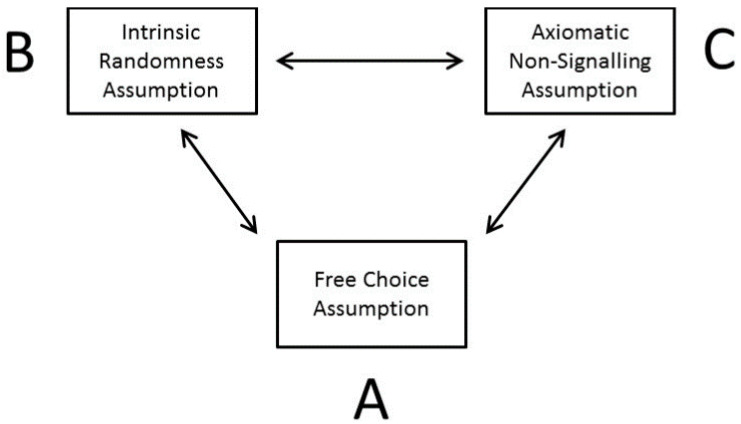
Illustration of the irreducible interdependency of basic assumptions that are implicit in standard interpretations of orthodox quantum mechanics (adapted from Walleczek and Grössing [27,83]). (**A**) Free choice assumption, (**B**) Intrinsic randomness assumption, and (**C**) Axiomatic non-signaling assumption. Importantly, the validity of interpreting the non-signaling theorem as a foundational theorem, or axiom, for quantum mechanics, i.e., one which would imply strict indeterminism as the only viable option for interpreting quantum theory, depends on the independent validity of assumptions (**A**,**B**). However, neither assumption (**A**) nor assumption (**B**) can be confirmed independently if the possibility of ‘free choice’ depends on the existence of a process that is intrinsically random and vice versa (compare Figure 1). Therefore, for example, the observation of EPR-type nonlocal correlations in the laboratory does not represent empirical proof for the indeterministic nature of the locally observed measurement outcomes, if that proof relies on the employment of an axiomatic non-signaling theorem (for more details see Walleczek and Grössing [27]).

Standard no-go theorems, such as Bell’s theorem [28] or, again, the Conway-Kochen free-will theorem [74,75] fail to account for this contradiction within the orthodox view, which is implied by super-indeterminism (see the legend to Figure 1). Therefore, such no-go theorems, i.e., the theorems claiming the impossibility of particular ontological propositions, imply conclusions of debatable value against the validity of deterministic quantum theories. For example, John Bell recognized the shortcomings himself regarding his own (no-go) theorem in view of an axiomatic interpretation of the non-signaling theorem, and he later adjusted his views [78,79,80,81,82]. For a detailed analysis of Bell’s evolving positions—from an axiomatic to an effective non-signaling constraint—see Walleczek and Grössing [27]. Concluding, the simple concept of super-indeterminism (Figure 1) explains why the free choice assumption of the experimenter agent in selecting measurement settings does not imply the necessary rule of the standard, i.e., axiomatic, non-signaling theorem (for details see Figure 2).

#### 5.1.1. On the Impossibility of Proving the Truth of Quantum Indeterminism

Long-running arguments against the possibility of deterministic, quantum-ontological approaches are increasingly criticized as falling short of their stated aims, in particular those based upon the free-will theorem and the non-signaling constraint as an axiom (see Figure 1 and Figure 2). Importantly, it is widely accepted that quantum indeterminism in the form of actual or objective chance can neither be proven by empirical tests nor by mathematical reasoning (e.g., [84]). However, what might be provable instead is the objective unpredictability of individual quantum measurement outcomes, as defined, for example, by a formal theorem such as Turing incomputability (see Section 5.3 and Section 6). Again, “indeterminism” captures a metaphysical assumption about how nature really is—prior to any formal theorizing. Furthermore, an empirical proof of indeterminism is out of reach, likely always, as a final loophole-free test seems to be a logical impossibility (compare Figure 1).

Finally, for a long time, because of the widespread belief that constraints such as absolute unpredictability, free will, nonlocality, non-signaling, or contextuality, could be compatible only with quantum indeterminism, any deterministic approaches to quantum theory have met with little interest by the mainstream of quantum physics, except often in reference to the perceived implausibility of the SW ontological quantum approaches (compare Section 2). This lack of interest has long been due to the near exclusive use—in the mainstream discourse on quantum foundations—of definite, non-contextual ontological assumptions, i.e., those that are consistent only with the classical, metaphysical assumption known as direct or naïve realism. As was mentioned before, the proposed “elements of reality” in the argument by Einstein et al. [38] represent, of course, entirely non-contextual ontic states in agreement with the classical metaphysics of naïve realism; there, the “elements” merely reveal their own “intrinsic”, already given, properties at the moment of their measurement.

As was reviewed in Section 3.1, any non-contextual, measurement-independent ontology, such as naïve realism, is wholly incompatible with the measurement predictions of orthodox quantum mechanics [39]. In recent years, however, new research has been pushing the frontiers of ontological possibilities beyond naïve realism, such as in the form of relational ontologies (e.g., Esfeld [85]), time-symmetric ontologies (e.g., Leifer and Pusey [12]), including unconventional causal structures such as retrocausality (e.g., Sutherland [29], Price [30], Wharton [13,31], Price and Wharton [32]). In addition, there has been a revival of interest in the nonlocal and contextual ontologies related to dBB-theory [1,2,3,4] and Bohmian mechanics [5,6,7,8], which are ontological propositions that posit the fundamental interconnectedness, instead of the intrinsic randomness, of the physical universe (e.g., Walleczek and Grössing [86]).

The focus of the subsequent Section 5.2 and Section 5.3 will be an assessment of the continuing possibility of ontology and determinism in quantum theory in relation to the experimenter agent. Specifically, what is sought is a scientifically based notion of “determinism without pre-determination” [60,86]. Next, Section 5.2 presents the traditional option for quantum mechanics in a globally deterministic universe.

### 5.2. Ontological Quantum Mechanics: “Effective Ignorance in Global Determinism”

**Figure 3:**
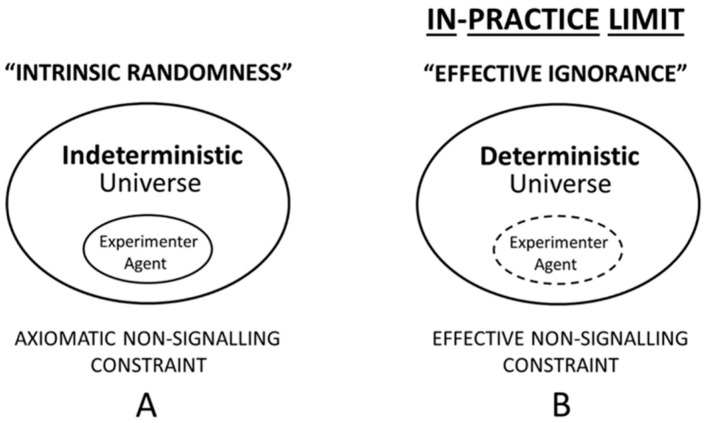
Agent inaccessibility as a function of (**A**) Intrinsic randomness versus (**B**) Effective ignorance (adapted from Walleczek [60]). Intrinsic randomness represents the orthodox interpretation of quantum mechanics, which is universal indeterminism. There, the presence of the experimenter agent introduces an apparent metaphysical dualism between agent and world (see the main text for additional explanations), which is indicated by the *closed* line that encloses the presence of the experimenter agent (Figure 3A). By contrast, in universal or global determinism, agents and the physical universe are subject to the same fundamental determinism, whereby, there, the experimenter agent is an integral element of the physical universe, i.e., agent and universe together constitute a lawful, physical continuum (e.g., Szilard [69]), as is indicated by the *open* line (see Figure 3B). In this picture, the experimenter agent constitutes an entity possessing distinct ‘epistemic’ as well as ‘agentic’ properties (for definitions see Section 4.3). For a detailed explanation of an axiomatic (Figure 3A) versus an effective (Figure 3B) non-signaling constraint—in the context of Bell’s nonlocality theorem—consult Walleczek and Grössing [27]. Briefly, an axiomatic non-signaling constraint (see also Figure 2) is compatible with the violation of measurement outcome independence, which is the standard violation in the context of orthodox quantum theory; by contrast, an effective non-signaling constraint is thought to be compatible with the violation of setting or parameter independence (Shimony [87]), which is the standard violation in the context of an ontological quantum mechanics such as dBB-theory in a universally deterministic universe (Section 3.3).

Instead of the metaphysical assumption of intrinsic randomness (Figure 3A), an ontological quantum mechanics opts for an alternative approach to explain the origins—in a globally deterministic universe—of experimentally observed quantum randomness. That is, ontological approaches typically seek an agent-dependent explanation based upon the unpredictability of individual measurement outcomes as a function of an epistemic limit, which—in the present analysis—is introduced as ‘effective ignorance’ (Figure 3B).

Importantly, the approach towards an “effective randomness”—by way of the concept of ‘effective ignorance’—is an option that can be consistently adopted if agent and universe are not metaphysically separated entities as suggested by the *open* line in Figure 3B (for details see legend to Figure 3). This is in contrast to the orthodox view shown in Figure 3A, where the agent stands in a physically isolated (quasi-transcendent) position towards the rest of the physical universe. For explanation, in the orthodox interpretation of quantum indeterminism, the agent is presumed to be capable of somehow initiating new cause-effect chains “out of nothing”, e.g., in violation of Leibniz’ Principle of Sufficient Reason (compare Section 5.1). This extra-physical agentic power is reminiscent of Maxwell’s demon-agent who was—falsely—thought to be unconstrained by the Laws of Nature, such as by the Second Law of Thermodynamics (see Section 4.2). This isolated, or dualistic, notion of agency in the orthodox picture is indicated by the *closed* line in Figure 3A (for details see legend to Figure 3).

The essential point of ‘effective ignorance’ is the following (Figure 3B): If assuming that the complete initial conditions of some deterministic system could be obtained, then the exact prediction of outcome states is possible—at least in principle. An example is a computer-generated pseudorandom bit sequence that becomes fully predictable once the (random) seed, i.e., the initial condition, as well as the algorithm, which is used to generate the bit sequence from the seed, is known to the scientific agent. By analogy, having complete knowledge of initial conditions, the properties of a (deterministic) quantum state could be computed, e.g., for the purpose of prediction and control, even if possessing finite computational resources only. Significantly, in the case of effective ignorance—when discrete events are finite—while access to initial conditions (compare the “seed” above) is technologically impractical, there exists, however, no *formal* limit that fundamentally constrains access to the complete initial state. For explanation, the definition of finite resources includes the whole universe as a finite resource, which—again—imposes an in-practice, effective limit, but not an in-principle, objective limit. In summary, the notion of “effective” quantum randomness as a result of the weak epistemic option is—at least in principle—computable by a Turing machine, even if the whole universe is to be recruited as a super-computational resource to achieve quantum predictability.

#### 5.2.1. Understanding John Bell’s Concept of “Free Variables” for Quantum Mechanics

The weak epistemic option of effective ignorance is consistent with, and explains, Bell’s own proposal of effectively “free variables” [79]. “I would expect a serious theory to permit… ‘pseudorandomness’ for complicated subsystems (e.g., computers),” Bell [65] suggested “…which would provide variables sufficiently free for the purposes at hand.” In addition, Bell provided the following explanation [79]:

“Consider the extreme case of a ‘random’ generator which is in fact perfectly deterministic in nature—and, for simplicity, perfectly isolated. In such a device the complete final state perfectly determines the complete initial state—nothing is forgotten. And yet for many purposes, such a device is precisely a ‘forgetting machine’. A particular output is the result of combining so many factors, of such a lengthy and complicated dynamical chain, that it is quite extraordinarily sensitive to minute variations of any one of many initial conditions. It is the familiar paradox of classical statistical mechanics that such exquisite sensitivity to initial conditions is practically equivalent to complete forgetfulness of them.”

This in-practice limit, which Bell [65,78,79] had argued for, does not, however, deny the theoretical possibility that the evolution of a deterministic system could be (computationally) predicted—at least in principle—if it *were* possible to access and determine “the complete initial state” [79]. By contrast, under the assumption that there exists a *fundamental* limit on computability and agent knowledge about the initial state (compare Section 5.3) that theoretical possibility would be denied also. Although Bell did mention ‘deterministic chaos’ in the context of ‘pseudorandomness’ [65], he did *not* propose that chaotic dynamics may represent a limit in any *fundamental* sense. On that specific point, the present work revises the conclusions of an earlier discussion of Bell’s effectively free-variables concept [27,60]. 

By relying on an additional principle, sometimes the powers of the weak option of effective ignorance are sought to be enhanced (e.g., Aharonov et al. [88]): the Uncertainty Principle prevents the simultaneous determination with arbitrary precision of, e.g., particle properties, thereby failing to characterize the relevant initial conditions for the same instant of time. However, the concept of ‘uncertainty’ is an operational, epistemic notion also, and the physical foundations of the Uncertainty Principle also remain to be identified (e.g., Rozema et al. [89]). Summarizing, the weak epistemic option represents an instance of subjective agent-inaccessibility, because that option depends upon the incomplete state of knowledge of the experimenter agent, i.e., upon an “uncertainty”, about the physical universe, including about initial conditions. However, note that even if the entire universe were available as a super-computational resource, then the presence of a black-hole singularity, for example, might render impossible even the purely theoretical prospect—in the weak epistemic option—of the cosmic computability of an individual quantum measurement outcome.

#### 5.2.2. Criticizing the Weak Option Interpretation

The weak option described above has often been criticized on the grounds that quantum randomness cannot possibly be a function of merely some in-practice limit on agent knowledge (Figure 3B). That skeptical position is echoed, for example, by Bub [35], who noted that quantum probabilities that describe the “nonlocal probabilistic correlations that violate Bell’s inequality” must be “intrinsically random events”, and that these probabilities “do not quantify incomplete knowledge about an ontic state (the basic idea of ‘hidden variables’).” For a counterpoint to Bub’s skeptical position, consult, for example, Figure 1 and Figure 2 in the present article (Section 5.1). Finally, Bub [35] also reaffirmed the popular position that this very fact in particular “…means that quantum mechanics is quite unlike any theory we have dealt with before in the history of physics.” 

Indeed, the perceived uniqueness of quantum mechanics, and it is supposed ‘weirdness’, is often cited as an “explanation” for strange or surprising features that are encountered in quantum studies involving single-particle observations. Specifically, concepts such as superposition (e.g., Schrödinger’s cat) and objective chance (i.e., intrinsic randomness)—in the form of objectively unpredictable measurement outcomes—are presumed to operate exclusively in the domain of the quantum, but never in the classical domain. However, what equally ‘weird’ phenomena may be produced as part of entirely classical systems? One example is the notion of ‘undecidable dynamics’ in classical systems as a function of self-referential systems dynamics. The present work introduces self-referential dynamics as a novel explanation that might underpin the physics of agent inaccessibility (see Section 5.3). This third and final option counters the idea that what distinguishes a quantum from a classical system is the capacity to generate objectively unpredictable outcomes.

### 5.3. Ontological Quantum Mechanics: “Objective Ignorance in Global Determinism”

The hypothesis that objective ignorance, as opposed to effective ignorance, can be the source of the unpredictability of individual quantum events in a deterministic system, represents the strong ontological option for explaining the physics of agent inaccessibility. Specifically, it had previously been proposed that agent inaccessibility in ontological quantum mechanics might be due to the limit that “…self-referential processes may generate physical observables whose values are universally uncomputable, i.e., their computation would require an infinite amount of computational resources” (Walleczek [60]). Briefly, the key feature of a nonlinear dynamical process called ‘self-referential’ is that a system output becomes a new input for the system within the same system (e.g., Walleczek [90]). In *dynamical chaos*, the constant action of feedback loops (recursive processes) is responsible for the generation of the chaotically evolving dynamics. In physical systems that can be characterized by *undecidable dynamics*, self-referential, recursive processes are, again, responsible for the objective unpredictability of outcome states. Importantly, the presence of self-referential dynamics (see Table 1 below) can be identified both in concrete physical systems as well as the computational models that describe them.

**Figure 4:**
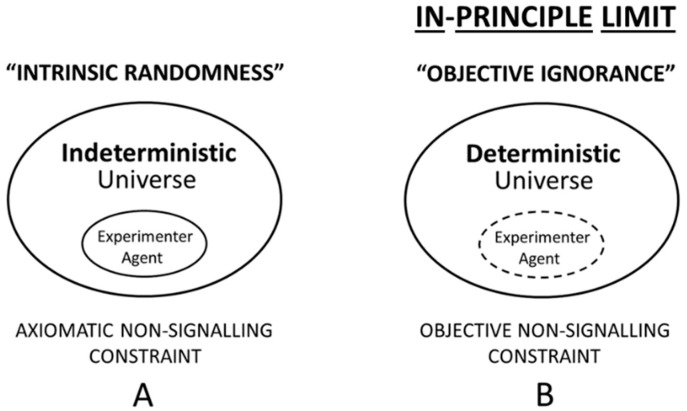
Agent inaccessibility as a function of (**A**) Intrinsic randomness versus (**B**) Objective ignorance (adapted from Walleczek [60]). Intrinsic randomness represents the orthodox interpretation of quantum mechanics, which is universal indeterminism (see legend to Figure 3 for an explanation of the nature of the experimenter agent). Objective ignorance, by contrast, advances the alternative proposal that quantum mechanics in a universally deterministic universe (i.e., global determinism) could account for (objective) quantum unpredictability as defined by an in-principle limit (Figure 4B). Please note that a prior report referred to a related proposal by the term ‘intrinsic complexity’ [60] due to the fact that such an option is available for complex systems dynamics. An objective non-signaling constraint, which is proposed here as an option that may underlie the non-signaling theorem of quantum mechanics, is equally governed by an objective, in-principle constraint; that is, the capacity for operational control by the experimenter agent (for definition see Section 4.3) of, for example, time-symmetric, or nonlocal, ontic influences, or information transfers, is formally and objectively limited by the unavailability to the agent of either (i) infinitely precise knowledge about (time-symmetric) initial conditions, or (ii) infinite computational, or generally technological, resources, or a combination of (i) and (ii). For an overview, see Table 1.

The strong option based upon fundamental uncomputability of outcome states—as a necessary and sufficient criterion for objective ignorance—is illustrated in Figure 4B. This proposal is contrasted with the orthodox position of intrinsic randomness shown in Figure 4A. Importantly, two different types of self-referential dynamics are currently known to support the concept of formal uncomputability—dynamical chaos and undecidable dynamics; each type posits the lack of *infinite* resources as a fundamental limit on computability (see Table 1). The question of the physical plausibility of the notion of formal uncomputability in the account of the objective unpredictability of quantum processes in nature will be discussed in Section 6.

A key distinguishing feature of the concept of objective ignorance—in contrast to that of effective ignorance—is the following (Figure 4B): Even if assuming that the *complete* initial conditions of some deterministic system could be obtained, then the exact prediction of outcome states is still impossible—even in principle. That is, in the option of objective ignorance (Figure 4B), the lack of *infinite* computational resources as a criterion places an *objective* limit on the experimenter agent as a function of undecidable dynamics (see Table 1), which, as Bennett [91] put it, is dynamics that is “…unpredictable even from total knowledge of the initial conditions”. This type of objective unpredictability is exemplified also in the halting problem for Turing machines, with the essential point being that Turing machines “…are unpredictable”, as Moore [92] noted, “even if the initial conditions are known exactly”. 

A second key distinguishing feature which is covered by the strong option of objective ignorance, but not by effective ignorance (Section 5.2), concerns the emergence of dynamical chaos in physical systems. Importantly, due to the theoretical impossibility of gathering information with *infinite* precision about the initial state from which evolves a dynamically chaotic system, an *objective* limit is imposed on the computability of the system’s outcome states. For explanation, note that *arbitrarily* small differences in initial conditions may generate strongly divergent outcome states in computational models of dynamical chaos (see Table 1). 

Because the strong option is also a knowledge-constraining option, the term ‘ignorance’ has been retained as part of the present proposal of an AIP for quantum mechanics. However, in contrast to effective ignorance, in the concept of objective ignorance, agent knowledge is not incomplete in the sense that gathering more information about initial conditions, or amassing more computational power, might eventually lead to complete knowledge and total predictability. Instead, an in-principle limit guarantees the incompleteness of agent knowledge, and therefore the agent’s inability to control and predict even a single quantum measurement outcome is ensured (see Table 1).

Therefore, the concept of objective ignorance represents an instance of *objective* agent-inaccessibility, which—obviously—is a more restrictive notion than *subjective* or *effective* agent-inaccessibility. Accordingly, the difference between the *effective* non-signaling constraint (Figure 3B) and the *objective* non-signaling constraint (Figure 4B) is that the latter constraint adopts a fundamental, and not a practical, limit on complete agent access towards an ontic state λ, and towards quantum information transfers, in ontological quantum mechanics in general. For example, this holds true for (SW) quantum ontologies that are locally time-symmetric [12,13,14,15,16,29,30,31,32], locally time-asymmetric [45,46,47], or strictly nonlocal [1,2,3,4,5,6,7,8]. Finally, the here proposed principle (AIP) is fundamental in the sense that a Turing oracle only could predict the exact value of an individual outcome state as a function of physical systems and computational model evolution. The strong option of objective ignorance (Figure 4B) might represent a fundamental principle by which nature prohibits access to the experimenter agent in the quantum regime. In the subsequent Section 6, a selection of available views and results are reviewed briefly which may support the present proposal for an AIP based upon the concepts of objective unpredictability, undecidability, and uncomputability.

## 6. In Search of Incomputable Nature: Quantum Reality and Quantum Randomness

The use of computational concepts and terminology in the search of the origins of the observed randomness in quantum systems, in combination with the recent “ontological turn” in quantum foundations (see Section 1), offers a new pathway towards exploring the physics of agent inaccessibility. In regard to the radical concept of incomputability in nature, one of its pioneers, S. Barry Cooper, once remarked—in reference to the puzzling features of nonlinear emergent states and chaos in nature—that “…many of the troublesome problems can be placed in a helpful explanatory context…” if one “…admits the possibility that the Universe is deeply imbued with incomputability and its mathematics” [93]. 

How realistic is the proposal that notions such as computability and uncomputability are relevant for physical laws, i.e., for the laws that explain the behavior of concrete micro-physical systems in nature, including those that are quantum-based? For example, Lloyd [94] has recently advanced the position that “…uncomputability is ubiquitous in physical law”, and that this is a natural consequence, he argued, of the fact that many “…physical systems are capable of universal computation”. Importantly, “…it is difficult to find an extended system with nonlinear interactions that is not capable of universal computation”, he explained, “…given proper initial conditions and inputs”. Furthermore, he argued that there may be special cases when “…quantum systems that evolve according to nonlinear interactions are capable of universal computation”, which yields the path-breaking possibility that “…the halting problem arises in the computation of basic features of many physical systems” [94]. 

Crucially, therefore, the concepts of uncomputability and undecidable dynamics [18,19,20,21,91,92,93,94] may have far greater significance to physics, and to the limits of science in general (compare Section 3.2), than—merely—as a concept that describes an abstract problem in recursive logic. For example, Rucker [95] has also argued that “…we should be able to find numerous examples of undecidability in the natural world”. Consequently, the formal concepts of undecidability and uncomputability may challenge the need for the (unprovable) metaphysical assumption of indeterminism as an explanation for the objective unpredictability in quantum systems. For example, Cubitt et al. [96] offered a physical model demonstrating the notion of objective unpredictability, not however as a function of quantum indeterminism, but due to self-referential, undecidable dynamics operating in the quantum regime (compare Table 1 in Section 5.3). 

### 6.1. Computational Approaches to Quantum Theory Invoking Nonlinear Interactions

The method of conceptualizing, or even explaining, the physical universe as a (quantum) computational process has a long history, and for recent overviews, see, e.g., Cooper and Soskova [97] and Fletcher and Cuffaro [98]. For example, in relation to quantum mechanics, researchers such as ’t Hooft [45,71] and Elze [46] have long promoted the idea that the probabilistic aspect of quantum physics does not necessarily have to contradict its possible algorithmic nature as demonstrated in work with quantum cellular automata. Generally, cellular automata (CA) can present models of the physical world and for the following discussion the equivalence of Turing machines and CA is assumed. “It is conceivable that the physical processes described by the laws of nature never come to an end”, Franke [99], for example, remarked, and that in adopting a CA-simulation of the physical world, “…we are simulating the behavior of a cellular automaton which runs deterministically, but is not computable.” Franke [99] emphasized that in such a model—therefore—the apparent randomness in the world might be due to an “…equivalent of chaos as understood in dynamical chaos theory, which as we know, is not based on actual chance, but on non-computability”. For explanation, Franke [99], in that quote, refers to ‘actual chance’ as denoting the standard indeterminism of orthodox quantum theory. By contrast, the non-computability stems from the fact that the possession of knowledge about the initial conditions of a dynamically chaotic process is not possible with infinite precision, which imposes a fundamental, in-principle limit on computability (see Table 1 in Section 5.3).

Very recently, the potential power of the approach that combines the notion of universal computation with unconventional *ontological* propositions has also been noted, for example, by Koberinski and Müller [43]. They considered the kind of information-theoretic properties of quantum theory “…which are directly linked to the possibility of having a universal computing machine, like the quantum Turing machine”, which is “…in principle able to simulate the time evolution of any physical system”. These authors have proposed that the “…notion of ‘universal computation’… is powerful enough to uniquely determine the state space, time evolution, and possible measurements (and thus also other properties like the maximal amount of non-locality) of quantum theory.” Again, however, as was emphasized by Lloyd [94], any computational interpretation of quantum systems might give rise to uncomputable elements, i.e., undecidable outcome states, which—within the constraints of a universal Turing machine—may therefore yield, again, a fundamental limit on agent-quantum access and predictability regarding the calculation of exact outcome values or individual ontological properties. One specific model of undecidable dynamics operating in the quantum regime was mentioned above [96]. Besides the notion of objective or fundamental *uncomputability*, how might the notion of the indefinite, contextual, or relational, *ontology* (for details see Section 3.1) enter the picture of the information-theoretic approach towards a quantum reality?

### 6.2. Quantum Ontology and the Information-Theoretic Paradigm in Quantum Mechanics

As was described in Section 5.1.1, novel ontological possibilities beyond naïve realism are increasingly considered as a basis for quantum mechanics, given that indeterminacy proofs are impossible. This includes relational ontologies such as ontic structural realism (e.g., [85,100]), locally time-symmetric ontologies (e.g., [12]), including unorthodox causal structures such as retrocausality (e.g., [29,30,31,32]). In the pursuit of possible *ontological* features of quantum mechanics, Koberinski and Müller [43] have also speculated about the presence of a relational ontology as part of a future construction of quantum theory, in particular, in reference to the proposal of ontic structural realism [85,100]. They have acknowledged that while “…the information-theoretic reconstructions… do not typically tell us what quantum states are, or what is really going on in the world when we perform a Bell experiment, for example”, the possibility might be considered of an “…ontology of structural relations in some sense—simply of the relational structure uniquely picked out by the information-theoretic postulates…”, which is an approach, they suggested, that “…does not rule out the possibility of discovering a constructive successor to quantum theory, in particular since ontological stability across theory change is a characteristic of ontic structural realism.” The combined computational-ontological research strategy, such as the one described above, may chart a new course also towards understanding the original HV-concept in Bohm’s ontological quantum theory (Section 3.3). That is, the application of ideas such as dynamical chaos and undecidable dynamics (see Table 1), to quantum ontology in dBB-theory and Bohmian mechanics, may in the future allow a new understanding of the variables traditionally called ‘hidden’ as uncomputable variables (compare Section 3.3).

### 6.3. Could Hidden Variables Represent Uncomputable Variables Such as Turing-Incomputable Variables?

Following the above analysis, the HVs of original dBB-theory may not only be ‘hidden’, and uncontrollable, in the familiar sense of the weak option known as ‘effective ignorance’ (Section 5.2); instead, the HV-concept might represent a case of uncontrollability and unpredictability as a function of the strong option involving nonlinear relations as described by the concept of ‘objective ignorance’ (Section 5.3). That proposal suggests the presence of a fundamental limit on agent inaccessibility in dBB-theory based upon the interpretation of the HV-concept as, for one speculative possibility, a Turing-incomputable variable (TIV). At a minimum, for starters, the proposal of TIVs in an ontological quantum mechanics, such as dBB-theory [1,2,3], would require—in the *constructive* approach, at least—the presence of nonlinear, self-referential interactions as part of the ontology of a quantum theory, i.e., an ontology that is compatible with emergence and chaos theory (see Table 1). Where in the Bohmian approach could that be found? Could Bohm’s theory manifest self-referential, chaotic behavior in a way similar to that seen in some constructions of an emergent quantum mechanics, which implements self-referential dynamics as a basic resource also? 

The original writings of Bohm and Hiley [3] reveal that the nonlinear perspective on the quantum state in Bohm’s theory was evident already 25 years ago: “The general behavior described”, Bohm and Hiley [3] wrote, “…is similar to that obtained in the study of non-linear equations whose solution contain what are called stable limit cycles”, whereby, however, the “…difference from the usual kind of non-linear equations is that for each stable motion we have a whole set of possible limit cycles rather than just a single cycle. Each quantum state thus corresponds to a different set of limit cycles and a transition corresponds to an orbit going from one of these to another”. Importantly, quantum state transitions, as the authors further explained, happen at “…bifurcation points dividing those orbits entering one channel from those entering another. Near these points, the motion is highly unstable and, indeed, chaotic in the sense of modern chaos theory” [3]. To mention only one new example: Work by Tzemos et al. [101] has described the origins of chaos in a mathematical model of a generalized Bohmian quantum theory. To be sure, there are additional reports that Bohmian trajectories could be chaotic and that chaotic dynamics could be the source of ‘quantum relaxation’ in Bohmian mechanics (e.g., References [102,103,104]). 

Work such as the above may pave the way towards conceptualizing the HV as an (effectively) uncomputable variable, or possibly even a TIV should, e.g., evidence for undecidability emerge in a future quantum-theoretic construction (see Table 1). Next, one topic of debate has long been the potential risk of violating the non-signaling condition of quantum mechanics as a function of the intrinsic nonlocality of ontological quantum theories such as dBB-theory (compare Section 3.3). For prior work which defined an *effective* non-signaling constraint for ontological quantum mechanics based upon an analysis of the concept of free variables by John Bell (see Section 5.2), consult Walleczek and Grössing [27]. Here, the concepts of an *effective* (Section 5.2.) and of an *objective* (Section 5.3) non-signaling constraint will be discussed briefly in the context of approaches considering computational constraints towards fashioning an understanding of the non-signaling theorem. 

### 6.4. The Non-Signaling Theorem and Effective versus Objective Computational Constraints

Bendersky et al. [63] have implemented a computational protocol to assess whether, or not, the nonlocal features associated with results from EPR-type quantum correlation experiments could be used to communicate messages between two space-like separated locations—in apparent violation of the non-signaling theorem. That study concluded that this is impossible because the “…computability of results imposes a strong limitation on how nature can behave if it only had computable resources to generate outputs for the experiments.” Central to that conclusion is, of course, the *standard* assumption that computational (Turing-type) processes “…cannot generate random sequences”, and that therefore, as Berendsky et al. [63] have added “…we need to accept the existence of truly unpredictable physical processes.”

Significantly, Berendsky et al. [63] concluded with the message that their findings are not in “…conflict with the different interpretations of quantum mechanics”, and they further noted that in “…the Copenhagen interpretation, the measurement process is postulated as random, whereas, for example, in Bohmian mechanics, it is deterministic but the initial conditions are randomly distributed and fundamentally unknowable.” For quantum theories operating in a universally deterministic universe (see Figure 3 and Figure 4), such as dBB-theory and Bohmian mechanics, the quantum randomness would be generated by uncomputable processes, whether they be *effectively* uncomputable (see the effective non-signaling constraint in Section 5.2), or (ii) *objectively* uncomputable in the strong sense of dynamical chaos and/or undecidable dynamics, e.g., in the form of Turing incomputability (see the objective non-signaling constraint in Section 5.3); only the strong option of objective ignorance in deterministic systems could entail objective or true unpredictability. However, the specific topic of self-referential dynamics in formal uncomputability (see Table 1) was not addressed in the work by Berendsky et al. [63], although these workers did make the important point that “...in Bohmian mechanics… the initial conditions are… fundamentally unknowable.” Previously, Islam and Wehner [105] had also suggested that quantum mechanics must entail the presence of (agent-inaccessible) uncomputable states as otherwise a violation of the non-signaling constraint would inevitably ensue, and these researchers noted that “…in any theory in which the Church-Turing principle holds, certain states and/or measurements are not available to us as otherwise any (approximate) no-signaling computation could be performed.” To employ the present terminology, in order (i) to prevent superluminal Shannon-type signaling in nonlocal quantum ontologies or, alternatively, (ii) to prohibit (future-to-past) retro-signaling in time-symmetric quantum ontologies, these “states and/or measurements” must be subject to an AIP as a fundamental principle in quantum mechanics.

### 6.5. Quantum Randomness and Turing Incomputability

How might the proposed link be explored further between Turing-incomputable processes and the problem of quantum randomness? On the one hand, a skeptic might argue against the notion of a successor to standard quantum theory, i.e., against the successful construction of a future quantum theory which could provide a *physical* account of quantum randomness. On the other hand, as was investigated in the present work, a new research movement is fast gaining traction which seeks to reanalyze, and explore again, the validity of ontological propositions for quantum mechanics (see Section 1). Could undecidable, Turing-incomputable processes be of significance for the research program towards an ontological quantum mechanics, including in the account of objective quantum unpredictability? Further evidence in favor of the plausibility of such a program has come forward in recent years. “Is quantum randomness Turing incomputable?”, asked Calude [106], and he described “…a procedure to generate quantum random bits that provably are not reproducible by any Turing machine”. Based on work that employed an operational version of the theorem by Kochen and Specker [39], the author suggested that quantum randomness might be the best evidence, so far, for the existence of a Turing-incomputable phenomenon in the natural world [106]. For a detailed analysis of that possibility, which posits the existence of value-*indefinite* observables in nature (compare Section 3.1), see Abbott et al. [40,41,42]. Given that a formal proof for quantum indeterminism is lacking in principle (see Section 5.1.1), the promise of a formal proof for the uncomputability of the observed randomness in quantum experiments both suggests and motivates the present proposal of an AIP for quantum mechanics (Section 1).

In summary, if the best available evidence for the true randomness of a sequence (that is generated by a quantum-based randomness generator) is the uncomputability of that sequence by a Turing machine, then this does not—necessarily—imply that the origins of that randomness is to be found in the metaphysics of quantum indeterminism. Consult Section 5.1 regarding arguments against the possibility of indeterminacy proofs, and Section 5.3 regarding the possibility of objective (true) unpredictability in fully deterministic systems (see Table 1). Given an AIP based upon objective ignorance, the following question remains unanswered at present: How to determine *empirically* whether the source of experimentally observed quantum randomness is either (i) ‘intrinsic randomness’ as in the orthodox position of Section 5.1 (Figure 3A), or (ii) ‘objective ignorance’ as in the strong option proposed in Section 5.3 (Figure 4B)? At present it remains unknown whether decisive experimental tests could be identified and performed. Until such tests might become available, the decision should be postponed between quantum indeterminism versus agent inaccessibility as a fundamental principle in quantum mechanics.

## 7. Conclusions

The question remains an open one as to whether agent inaccessibility in quantum experiments is either (i) due to metaphysical indeterminism or (ii) due to a quantum ontology of a form where the *exact* ontic state λ is either effectively or objectively uncomputable. The latter option is the basis of the present proposal for agent inaccessibility as a fundamental principle (AIP) in quantum mechanics. What is the ontological status of a fundamentally agent-inaccessible quantum state? The status is *indefinite* of the agent-inaccessible (“hidden”) ontic state (IOS) because only an infinite amount of measurement information, and/or access to infinite computational resources, might enable the exact prediction of a *definite* measurement outcome (DOS). Finally, the concepts of self-referential dynamics and formal uncomputability may represent key elements in a physical theory of agent inaccessibility. Instead of framing the 20th century quantum revolution as a radical shift from determinism towards indeterminism, this work has argued that—given the available scientific evidence—it is valid only to claim the following: the quantum revolution signifies the profound discovery of an agent-inaccessible regime of the physical universe.

## Figures and Tables

**Table 1:** Two types of self-referential dynamics are considered as a basis for the proposed physics of agent inaccessibility. For the proposal of an AIP as a fundamental principle in quantum mechanics (objective ignorance), the objective unpredictability of an individual measurement outcome as part of a typical quantum random sequence is a function of formal uncomputability; both, dynamical chaos as well as undecidable dynamics posit “infinity”—the lack of infinite resources—as a fundamental limit on computability. Regarding the limit of infinite precision detection in relation to the concept of formal uncomputability, note that—in computational predictions of chaotic dynamics—an *arbitrarily* small difference in initial conditions may lead to a vastly different future outcome state. Note also that the concept of undecidable dynamics underpins both computational irreducibility [18,19] as well as the halting problem in the Church-Turing thesis [20,21].

Self-Referential Dynamics	Formal Uncomputability
Dynamical chaos	Infinite precision detection of initial conditions is impossible in-principle
Undecidable dynamics	Infinite computational resources are unavailable in-principle
